# The North Italian innovative project for common psychiatric disorders: Evaluating the output of a treatment model of an outpatient clinic for anxiety and depression

**DOI:** 10.3389/fpubh.2022.1024857

**Published:** 2023-01-10

**Authors:** Ivano Caselli, Alessandra De Leo, Celeste Isella, Alessandro Bellini, Marta Ielmini, Camilla Callegari

**Affiliations:** ^1^Department of Medicine and Surgery, Section of Psychiatry, University of Insubria, Varese, Italy; ^2^Department of Medicine and Surgery, Section of Psychiatry, University of Pavia, Pavia, Italy

**Keywords:** anxiety, depression, outpatient, innovative project, psychiatric disorders

## Abstract

Depressive disorders were considered the first causes of disability worldwide as early as 2018. The outpatient clinic for anxiety and depression at the University Hospital of Varese represents a service that fully responds to the growing number of requests. Approximately 1,350 medical records have been opened from 2010 to December 2021. The most frequent presenting diagnoses included anxiety disorders (36.8%), severe stress and maladaptation syndromes (35.5%), and depressive episodes (18%). The outpatient clinic has proved to be a model with great impact on users offering a range of diagnostic and therapeutic offers responding to the requests of the community.

## Introduction and context

As stated by MacCarron et al. depressive disorders were considered one of the first causes of disability worldwide as early as 2018 (1, 2). Considering this scenario, the outpatient clinic for anxiety and depression at the University Hospital of Varese, ASST (Azienda Socio Sanitaria Territoriale). Sette Laghi, represents a service that fully responds to the growing number of requests for consultations for anxiety and depressive disorders. The innovative project “Common psychiatric disorders: Treatment in cooperation with general practitioners” is fully funded by the Lombardy region. In addition to anxiety and depressive disorders, the offer is extended to perinatal depressive disorders and work-related stress conditions. A psychological help desk for the prison police officers of the Prison Institute has been included since the beginning of 2020. Moreover, from the beginning of 2021, a systematic activity of psychiatric counseling was also launched for users of the gynecology department of the hospital offering psychotherapy sessions for patients with cancer disease. As Talevi et al. observed, the COVID-19 emergency then significantly amplified the emotional distress in the general population with a significant impact on mental health services (3). In fact, the outpatient clinic has been identified as the reference service for the treatment of COVID-19-related psychiatric disorders, according to the Lombardy region guidelines to provide both online and presence consultations, also maintaining in 2021, the offer dedicated to the hospital health workers. The psychiatrist interventions include diagnostic and assessment evaluations (also using rating scales such as the *Hamilton Depression Rating Scale* for depression or the *Brief Psychiatric Rating Scale* for symptoms evaluation), the prescription of psychopharmacotherapy (4, 5), the selection for sending to psychotherapy, the drafting of specialist reports, the proposal of a therapeutic program at the clinic itself, or the sending to the competent psychiatric service. The psychological interventions involve the use of psychotherapeutic techniques of the cognitive matrix for the hospital setting, support interviews, assessment and sharing/co-construction of therapeutic objectives, the assignment of “homework,” medium–long term monitoring visits, the evaluation of psychotherapy outcome, and evaluation tests (i.e., *Minnesota Multiphasic Personality Inventory*) (6). The innovative program provides specialist assessments to users sent by general practitioners or other specialists. All psychiatric and psychological sessions are exempt from fees. The clinic is open 5 days a week, and the staff consists of two psychiatrists, a psychologist, and an administrative secretary. All interventions are personalized and customized for each patient.

## Key programmatic elements and results

The project provides an effective diagnostic and treatment framework, as well as prevents the exacerbation of even more disabling psychiatric disorders. In this sense, the clinic, inserted in the healthcare context of the psychiatric unit, is in continuity with the other mental health centers as well as with the psychiatric ward. The territorial users of the Varese area were privileged, but, as a regional project, users from all over Lombardy are welcomed. Currently, the wait for a first psychiatric evaluation is approximately 30–45 days, and the wait for a first psychological consultation is 2 or 3 weeks. The organization makes it possible to greatly reduce the waiting lists for a first psychiatric visit and a first psychological evaluation. Approximately 1,350 medical records have been opened from the end of 2010 to December 2021. The first visits were ~150 per year, and the annual services provided were ~2,000. In the year 2016, the first visits were 73, and the follow-up visits were 1,439. From 2017, the total number of visits has resumed being 1,800 on average. In 2020, due to the COVID-19 pandemic, there was a reduction in the total number of visits to 1,060, with 77 first visits. In the year 2021, 146 first visits and 2,046 services were carried out. Of 361 active patients, 90 were men and 271 were women. The average age was 51 years, and the most represented age groups were those between 45 years and 55 years (118 patients) and between 56 years and 65 years (82 patients). There were 211 online interventions. One hundred eighty-four patients followed a psychotherapy program. At the first consultation, ~25% of patients were already under treatment by psychologists, and ~10% were already in medication treatment set by general practitioners or during an emergency room consultation. In 31% of cases, the patient was offered a psychological path combined with psychiatric control interviews without the prescription of any medication. The remaining percentage was offered pharmacotherapy often combined with psychological sessions. The most frequent presenting diagnoses (according to the ICD-10 criteria, International Classification of Diseases, 10th Edition) are shown in the table below (7) (Table 1).

**Table 1:** Distribution of the diagnosis in 2021 (*N* = 361).

Diagnosis (ICD-10^*^)	**Number of patients (%)**	**Subtypes**	**Number of patients (%)**
Depressive episode (F32)	65 (18%)	Mild depressive episode (F32.0)	7 (10.8%)
		Moderate depressive episode (F32.1)	26 (40%)
		Severe depressive episode without psychotic symptoms (F32.2)	11 (17%)
		Unspecified depressive episode (F32.9)	2 (3%)
		Subtype not reported (F32)	19 (29.2%)
Other anxiety disorders (F41)	133 (36.8%)	Panic attacks disorder (F41.0)	26 (19.5%)
		Generalized anxiety disorder (F41.1)	30 (22.6%)
		Mixed anxiety and depressive syndrome (F41.2)	56 (42.1%)
		Other mixed anxiety syndromes (F41.3)	6 (4.5%)
		Other specified anxiety disorders (F41.8)	8 (6%)
		Unspecified anxiety disorder (F41.9)	2 (1.5%)
		Subtype not reported (F41)	5 (3.8%)
Reaction to severe stress, and adjustment disorders (F43)	128 (35.5%)	Acute stress reaction (F43.0)	2 (1.6%)
		Post-traumatic stress disorder (F43.1)	0
		Adjustment disorders (F43.2)	22 (17.2%)
		Brief depressive adjustment reaction (F43.20)	27 (21.1%)
		Adjustment disorder with depressed mood (F43.21)	7 (5.5%)
		Adjustment disorder with anxiety (F43.22)	61(47.6%)
		With prevalent disorder of other emotional aspects (F43.23)	4 (3.1%)
		With mixed emotional and behavior disorder (F43.25)	3 (2.3%)
		Other reaction to severe stress (F43.8)	2 (1.6%)
Personality disorders (F60)	11 (3%)		
Other disorders	24 (6.7%)		

* ICD-10, International Classification of Diseases, Tenth Edition.

## Discussion

The clinic has provided for the improvement of communication with general practitioners through written reports and telephone or email communications. This may lead to an improvement in the early detection of distress and mild depression by general practitioners, and sending them to specialists in a short time, thus promoting screening in primary care (8). Qualitative analyses showed that the negative perception of the disease, the negative perception of treatment, the relevance of the social environment, and the doctor–patient relationship are crucial aspects in explaining the non-consulting of a general practitioner during a depressive episode. It has been shown that better information on depression and its treatments, and screening by primary care personnel would improve the treatment of a patient with depression (9, 10). The integration of the outpatient clinic into the hospital aims to reduce the stigma against psychological distress and to create a social climate sensitive to mental health problems. As can be seen from the data, a good percentage of cases are sent by private psychologists. In relation to this, the outpatient clinic aims at integration between the pharmacological intervention or counseling and the psychological one through telephone communications that occur regularly between the psychologists and the referring psychiatrists. On the other hand, a large percentage of patients are followed by both the psychiatrist and the psychologist of the clinic, and communication between these two figures is favored by frequent meetings on cases. Although clinical research has not yet conclusively demonstrated the superiority of combined therapy over single treatments, there is some evidence for depression (11).

The outpatient clinic for anxiety and depression of the university psychiatric unit is considered an innovative program funded by the Lombardy region since 2010. It has proved to be a model with great impact on users offering a range of diagnostic and therapeutic offers responding to the requests of the population, the interdisciplinary needs within the hospital, and the increase in depressive disorders in the community. The model has proved to be an innovative program due to its structural and organizational dimension in the Regional Health System.

## Data availability statement

The raw data supporting the conclusions of this article will be made available by the authors, without undue reservation.

## Author contributions

IC was the principal writer of the manuscript. AD assisted in writing the draft of the manuscript and gathered relevant data. AB provided significant editorial support. CI and MI assisted in developing the idea of the manuscript. CC developed the idea for the manuscript and supervised the final draft of the manuscript. All authors contributed to the article and approved the submitted version.
